# Supplementary material for: Prediction method for the reaction force of vehicle door sealing systems
Source: PLoS One. 2025 Nov 25;20(11):e0336802. doi: 10.1371/journal.pone.0336802 (PMC12646454; doi:10.1371/journal.pone.0336802)
Supplement: Attachment — Submitted filename: ANSWERS TO EDITOR(S) AND REVIEWER(S).docx [file pone.0336802.s003.docx]

**ANSWERS TO EDITOR AND REVIEWER(S)**

Dear Editor(s) and Reviewer(s),

Thank you very much for reviewing our manuscript titled “Prediction Method for the Reaction Force of Vehicle Door Sealing Systems (Manuscript ID: PONE-D-25-31882)”. The respected reviewer(s) have brought up some valuable comments and constructive remarks that helped us to significantly revise and improve our manuscript. While revising the manuscript, we have checked all the general and specific comments provided by the reviewers and have made the necessary changes to take account of their questions and comments. Furthermore, all sections of the manuscript including Title, Abstract, Introduction, Methods, Figures (graphs), Tables, Results and Discussion, Conclusion and References have been checked and revised thoroughly. The revised manuscript is believed to be clearer, more logical, and hence more likely to prevent error and confusion.

**Major revisions**

**C1-1:** The discussion on the research related to electric vehicle sound quality and motor noise should be removed, and the research scope should be focused on the sealing system and static closing force.

**A1-1:** Thank you very much for your suggestion. This study focuses on the simulation and testing of the sealing reaction force of the door system. The introduction section has removed the discussion on research related to electric vehicle sound quality and motor noise, while retaining the NVH content associated with the impact of static force on door closing/noise. The research will focus on the sealing system and static closing force. Thank you!

With the rapid electrification of the automotive industry, consumers' demand for a refined driving experience is increasing, which highlights the importance of vehicle NVH (Noise, Vibration, and Harshness) performance. Previous studies [1-3] have shown that the sound quality of vehicle door opening and closing has a significant impact on user perception and product satisfaction, and it is a key influencing factor of vehicle NVH performance. The reaction force of the sealing system is an important evaluation index for the sound quality of door opening and closing, and it directly affects this sound quality. Insufficient reaction force of the sealing system will weaken the sealing contact between the door and the vehicle body frame, leading to door vibration and dynamic sealing problems [4]; on the contrary, excessive reaction force of the sealing system will not only increase the door closing force and amplify the unlocking sound (thus reducing acoustic comfort) but also impose excessive mechanical stress on the lock system [5-9]. Reasonable prediction and control of the reaction force of the sealing system have become an urgent task for automotive engineers, who aim to maintain competitive advantages by improving user perception and satisfaction. This study intends to systematically analyze the reaction force of the NEV (New Energy Vehicle) door sealing system through simulation methods and propose innovative optimization strategies to improve the sound quality of door opening and closing. Wagner et al. [10] took the lead in conducting a nonlinear analysis of seal compression load. Wang Jin et al. [11] established a mathematical model based on EXCEL to calculate the door closing force. Essentially, this calculation method simplifies the door and the sealing system into a single, decoupled model, ignoring the interaction between the door and the seal, which leads to errors in the calculation results.

To overcome the above influencing factors, this paper proposes a multi-boundary coupling simulation method for the door sealing system. This method uses finite element modeling technology to establish a door-seal finite element model and maps the measured/virtual CLD (Closing Load Deflection) curve to the assembled vehicle FE (Finite Element) model. The model considers the deformation of the door and the seal under the flexible coupling effect, enabling accurate simulation of the seal reaction force under closing conditions. In addition, this study implements a seal CLD value optimization strategy through contribution analysis, laying a foundation for the optimization of reaction force and the structural design of the seal. Meanwhile, tests are conducted on three types of vehicles to verify the effectiveness and universality of the proposed method (see Pages 3 and 5).

**C1-2:** Method/model coherence (hyperelastic seals vs. CBUSH springs).

**A1-2:** Thank you very much for your suggestion. Regarding the simulation method of the seal strip, we have revised the inconsistent parts in the paper and unified the consistency of methods and models. This paper adopts method (b) to simulate the seal strip, using a one-way coupled nonlinear static analysis approach. The seal strip is simulated with 1D spring element CBUSH at intervals of 100 mm, and the tested/simulated CLD parameters of the seal strip are mapped to the CBUSH elements.

Process of mapping the CLD curve to the nonlinear force-displacement characteristics of CBUSH: In accordance with the domestic industry standard *Test Method for Compression Load of Automotive Seal Strips*, seal strip samples with a length of 100 mm are used for testing, and the loading direction is the compression direction of the seal strip. Therefore, the seal strip simulation also uses a spring element arranged every 100 mm. The CBUSH element is a spring-damper component that can simulate stiffness in six directions. The direction of the CBUSH element is determined according to the test compression direction. At the same time, the CLD curve properties are assigned to the CBUSH element, as shown in Figure 1.

Why this is acceptable: The CLD curve is a simulated/tested value. Through comparison between simulation and experiment, the error is within 27%—a range that falls within the tolerance of the target value (see Table 4 in Section 2.4 of the paper). Therefore, the simulation using simplified spring elements is acceptable.

Table 4 Simulation and Test Results of Cross-Section

| Location | | D0 Target Value N/100mm | D0 Simulation Value N/100mm | D0 Test Value N/100mm | Deviation |
| --- | --- | --- | --- | --- | --- |
| Door Edge Strip | Hinge Side | 5±2 | 4.38 | 4.24 | 3% |
|  | Transverse End Side | 5±2 | 3.69 | 4.24 | 13% |
|  | Door Lock Side | 5±2 | 3.96 | 4.24 | 7% |
|  | Threshold Side | 5±2 | 3.69 | 4.24 | 13% |
| Frame Seal Strip | Hinge Side | 3±1.5 | 3.01 | 4.15 | 27% |
|  | Transverse End Side | 3±1.5 | 3.67 | 4.15 | 11% |
|  | Door Lock Side | 3±1.5 | 4.32 | 4.15 | 4% |
|  | Threshold Side | 3±1.5 | 4.12 | 4.15 | 1% |

**C1-3:** Equations & units (Eqs. 1–3).

**A1-3:** Thank you very much for your suggestion. The description of the formulas in this paper was not clear enough, so the irrelevant and ambiguous parts have been deleted. Formula 1 is described in textual form as follows: The static closing force of the door is mainly contributed by three components: the seal strip, the door lock mechanism, and the buffer block.

Formulas 2 and 3 have been revised using the static equilibrium theory and re-numbered as Formulas 1 and 2.

After revision, an illustration and corresponding description have been added, as follows: As shown in Figure 2, the door rotates around the hinge axis. The seal strip can be divided into several consecutive segments, and the moment arm from the i-th segment of the seal strip to the hinge can be denoted as Li. Without considering door deformation and seal strip compression deformation, the total moment exerted by the seal strip on the hinge axis is:

$E_{\text{md}}=\sum_{i=1}^{N} \text{F}_{i}\text{L}_{i}$	（1）

In the formula,

Emd is the total moment, with the unit of N·mm (Newton-millimeters);

i is the total number of segments of the seal strip;

Fi is the force applied to the i-th segment of the seal strip, with the unit of N Newtons);

Li is the moment arm from the centroid position of the -th segment of the seal strip to the hinge axis, with the unit of m millimeters).

Then, according to the principle of moment equilibrium, the force at any point on the door relative to the hinge axis is:

$F=E_{\text{md}}/L_{s}=\frac{\left( \sum_{i=1}^{N} \text{F}_{i}\text{L}_{i} \right)}{L_{s}}$	（2）

Figure 2

Among them, Ls is the moment arm from any point on the door to the door hinge axis, with the unit of mm (millimeters). The static closing force of the door is mainly contributed by three components: door seal, door latch mechanism, and buffer block. (Page 6 and 7)

The terms in the equation, including Fj, Fmf, Fs, Fh, Fm, Fx, d0, F=Fm/100, L, Ls, and Ex(with uncertainty about "E"), have been redefined and described after removing the original formula symbols. For details, please refer to the derivation process.

Derivation of Equation 3:

As shown in Figure 2, the door rotates around the hinge axis; therefore, the hinge axis is taken as the reference position for moment calculation. When the door is closing, the seal strip is in a compressed state and exerts a pressure on the door. This pressure can be regarded as numerous small concentrated forces acting on various positions of the seal strip. For an object in a stationary state, the total moment of forces about any point is zero, i.e., ∑M=0. In other words, the resultant moment of the door reaction moment and the moment generated by the seal strip reaction force is zero.

The seal strip can be divided into several consecutive segments. The moment arm from the i-th segment of the seal strip to the hinge can be denoted as Li, and the compression load of the i-th segment is Fi. Without considering door deformation and seal strip compression deformation,

the moment exerted by the i-th segment of the seal strip is Ei=FiLi.

The seal strip and the door are simplified to a superimposable linear relationship. Therefore, the total moment of the seal strip about the hinge axis is:

$$E_{\text{md}}=\sum_{i=1}^{N} \text{F}_{i}\text{L}_{i}$$

Then, according to the principle of moment equilibrium, the force at any arbitrary point on the door relative to the hinge axis is:

$F=E_{\text{md}}/L_{s}=\frac{\left( \sum_{i=1}^{N} \text{F}_{i}\text{L}_{i} \right)}{L_{s}}$ That is, Equation 3.

**C1-4:** Boundary conditions, contact, solver settings.

**A1-4:** Thank you very much for your suggestions.

**Boundary Conditions**: All degrees of freedom of the mounting points at the joints between the upper and lower door hinges and the vehicle body are constrained, while allowing the hinges to rotate along the hinge axis. A Y-direction constraint is applied at the door handle position. CBUSH elements are placed every 100 mm to map the CLD values of the seal strip to the corresponding positions of the seal strip on the door.

**Solver:** The Nastran SOL106 solver is adopted for the nonlinear analysis.

In the simulation model of this paper, there is no need to consider the contact type, friction coefficient, or damping between the 1D spring elements and 2D door elements. The initial load increment is 0.1, with 10 load increment steps.

**Convergence Criterion**: Convergence is achieved when the tolerance is less than 1.0E-3, and an implicit solver is used.

The load refers to the force generated by the seal strip when compressed by the door. The loading positions are the contact areas between the seal strip and the door, with segmented loading applied at 100 mm intervals. The seal reaction force analysis does not take into account the deformation or pre-compression of the seal strip.

**Load Step Sensitivity**: As the load step size decreases gradually, the results tend to stabilize. For this model, the initial increment is set to 0.1; when the initial increment is adjusted to 0.05, the results remain stable without significant differences. This confirms that an initial increment of 0.1 is reasonable and the results meet the convergence requirements.

**C1-5:** Validation design & error analysis.

**A1-5:** Thank you very much for your suggestions.

For Table 7, corresponding labels and explanations have been added to the first row of the header, with revisions as follows:

The first column, originally "the average value ± standard deviation of three tests for the outer panel corresponding to the lock catch of three vehicle models, measured in accordance with the test specification", is revised to "Average Test Value".

The second column, which represents "the force value converted to the door handle position (simulation observation point) for easy comparison with the simulation results", is revised to "Reaction Force Converted to Door Handle Position".

The third column, originally "simulation reaction force value", is revised to "Sealing System Reaction Force".

The fourth column remains "Absolute Percentage".

Define the statistical measures used for your comparison: mean error ± standard deviation, with absolute percentage adopted as the exact error metric.

The error of the test instrument is ±1 N, and the operator variability is ±5 N.

A residual plot was generated based on the test results, as shown in Figure 3. The results indicate that the residuals are both positive and negative, fluctuating around the zero point—this confirms consistency in the data trend. The samples include Sedan A, SUV B, and SUV C. For each sample, the average value of three tests was compared with the simulation results, showing a maximum error of 9% and an average error of 7%.

**Chapter 4 is revised as follows:**

To verify the reliability and broad applicability of this analytical method, simulation and testing of the sealing system reaction force were conducted on three different vehicle models, including those with framed doors and frameless doors. The simulation analysis method is consistent with that described in Chapter 2.1.

The test equipment and scenario are shown in Figure 12. Tests were performed on the prototype vehicles to ensure that the prototype state was consistent with the designed state. The door was adjusted to the closed position, while the door lock remained in the unlocked state. A dynamometer (accuracy: ±1 N) was used to slowly apply a Y-direction thrust at the outer panel position corresponding to the door lock (30 mm from the edge of the outer panel) until the door was locked. The reading of the dynamometer at this moment was recorded as the door closing force, and the average value of three test results was taken as the final test result for recording.

The comparison between the simulation results and test results of the sealing system reaction force is presented in Table 7:

Table 7 Simulation and Test Results of Sealing System Reaction Force

| vehicle model | Average Test Value (N) | Reaction Force Converted to the Door Handle Position (N) | Sealing System Reaction Force (N) | Absolute Percentage Error APE | | | Mean Absolute Error MAE(N) | | Mean Absolute Percentage Error MAPE | | Root Mean Square ErrorRMSE（N） | |
| --- | --- | --- | --- | --- | --- | --- | --- | --- | --- | --- | --- | --- |
| Sedan A | 247±13 | 234 | 222 | | 5% | 25 | | 7% | | 18 | |  |
| SUV B | 252±13 | 210 | 226 | | 8% | 26 | | 8% | | 21 | |  |
| SUV C | 231±6 | 201 | 182 | | 9% | 49 | | 9% | | 20 | |  |

Figure 3

Sealing system reaction force tests were conducted on three vehicle models. Based on Equations (1) and (2), the average test values were converted to the reaction forces at the door handle position for comparison with simulation results. The Mean Absolute Percentage Error (MAPE) between the sealing system reaction force simulation and experimental results was 7%, with a maximum error of 9%. A residual plot was generated from the experimental results, which showed that the residuals were both positive and negative, fluctuating around the zero point. This confirms the consistency of the data trend and indicates that the analysis results are reliable. Therefore, the prediction method for door sealing system reaction force established in this study provides reliable methodological support for the design and optimization of sealing strips in the early stage of project development. (Pages 22 and 24).

**C1-6:** Contribution (CLD) analysis method.

**A1-6:** Thank you very much for your suggestions.

To evaluate the contribution of sealing strips to the sealing reaction force, the sealing strips were divided into regions. This division is mainly based on the consideration that door sealing strips have different interference amounts with the vehicle body at different positions, and the corresponding compressive loads also vary accordingly.

**Lateral End Side**	**Latching Side**

**Hinge Side**	**Sill Side**

**Figure 4**

From Equation 2, $F=E_{\text{md}}/L_{s}=\frac{\left( \sum_{i=1}^{N} \text{F}_{i}\text{L}_{i} \right)}{L_{s}}$ it can be seen that the magnitude of the reaction force is equal to the sum of the torques of the four regions divided by the moment arm. When the door structure is determined, the moment arm is a fixed value. The contribution analysis is conducted under the assumption that the door structure is fixed and the moment arm remains unchanged, mainly to identify the influence of the sealing strip's CLD (Characteristic Load Distribution) on the reaction force.

To evaluate the contribution of the CLD of the sealing strip in a single region to the reaction force, the sealing strips in the door lock, hinge, sill, and lateral end regions were respectively removed from the finite element model to calculate the reaction force. Other boundary conditions were kept unchanged, and a single-factor calculation was used to compare the changes in reaction force before and after removing the sealing strip in a certain region, thereby evaluating the reaction force contribution of the removed sealing strip segment.

Table 5-6 was obtained through the above calculation method. This table provides the simulation analysis results of the sealing strip contribution for three types of doors. It can be seen from the table that the contribution of each region of the sealing strip to the reaction force is consistent across the three vehicle models, indicating a certain universality of this rule. The simulation method and specific working condition description are detailed in Chapter 3.1, and the simulation method is universally applicable.

Table 5: Contribution of different regions of the door seal to the sealing system reaction force

| Vehicle Type | Door Strip（N） | Door Lock Side（N） | Roof Rail Side（N） | Hinge Side（N） | Sill Side（N） |
| --- | --- | --- | --- | --- | --- |
| - Sedan A | 94 | 65 | / | 2 | 27 |
| - SUV B | 115 | 73 | / | 4 | 38 |
| - SUV C | 88 | 49 | 19 | 2 | 17 |

Table 6: Contribution of different regions of the door frame seal to the sealing system reaction force

| Vehicle Type | Frame Strip（N） | | Door Lock Side（N） | Roof Rail Side（N） | Hinge Side（N） | Sill Side（N） |
| --- | --- | --- | --- | --- | --- | --- |
| Sedan A | | 128 | 82 | 9 | 2 | 36 |
| SUV B | | 111 | 72 | 2 | 6 | 30 |
| SUV C | | 92 | 45 | 25 | 2 | 20 |

**C1-7:** Figures & tables (labels, units, readability).

**A1-7:** Thank you very much for your suggestions. Units have been added to the post-processing contour plots of the finite element simulation analysis. The tables have also been standardized in the following format:

**FIG. 10.** Reaction force contour at the observation point

**FIG. 11.** Y-direction displacement contour of the door frame page18 19

Table 3: Material Parameters page 12

| Material | Elastic Modulus (MPa) | Poisson's Ratio | Density (KG/m³) |
| --- | --- | --- | --- |
| Steel | 206000 | 0.3 | 7830 |
| Spot Weld | 206000 | 0.3 | 7830 |
| Expansion glue | 1.63 | 0.41 | 1400 |

Table 1 Target Values of CLD for Door Frame Seal and Door Seal (Page 16)

| Position | | D0 Target Value (N/100mm) |
| --- | --- | --- |
| Door Seal | Hinge Side | 5±2 |
|  | Lateral End Side | 5±2 |
|  | Door Lock Side | 5±2 |
|  | Sill Side | 5±2 |
| Frame Seal | Hinge Side | 3±1.5 |
|  | Lateral End Side | 3±1.5 |
|  | Door Lock Side | 3±1.5 |
|  | Sill Side | 3±1.5 |

Table 4 Simulation and Test Results of Cross - Section (Page 17)

| Position | | D0 Target Value N/100mm | D0 Simulation Value N/100mm | D0 Test Value N/100mm | Deviation |
| --- | --- | --- | --- | --- | --- |
| Door Seal | Hinge Side | 5±2 | 4.38 | 4.24 | 3% |
|  | Lateral End Side | 5±2 | 3.69 | 4.24 | 13% |
|  | Door Lock Side | 5±2 | 3.96 | 4.24 | 7% |
|  | Sill Side | 5±2 | 3.69 | 4.24 | 13% |
| Frame Seal | Hinge Side | 3±1.5 | 3.01 | 4.15 | 27% |
|  | Lateral End Side | 3±1.5 | 3.67 | 4.15 | 11% |
|  | Door Lock Side | 3±1.5 | 4.32 | 4.15 | 4% |
|  | Sill Side | 3±1.5 | 4.12 | 4.15 | 1% |

Table 5: Contribution of different regions of the door seal to the sealing system reaction force

| Vehicle Type | Door Strip（N） | Door Lock Side（N） | Roof Rail Side（N） | Hinge Side（N） | Sill Side（N） |
| --- | --- | --- | --- | --- | --- |
| - Sedan A | 94 | 65 | / | 2 | 27 |
| - SUV B | 115 | 73 | / | 4 | 38 |
| - SUV C | 88 | 49 | 19 | 2 | 17 |

Table 6: Contribution of different regions of the door frame seal to the sealing system reaction force page20 and 21

| Vehicle Type | Frame Strip（N） | | Door Lock Side（N） | Roof Rail Side（N） | Hinge Side（N） | Sill Side（N） |
| --- | --- | --- | --- | --- | --- | --- |
| Sedan A | | 128 | 82 | 9 | 2 | 36 |
| SUV B | | 111 | 72 | 2 | 6 | 30 |
| SUV C | | 92 | 45 | 25 | 2 | 20 |

Table 7 Simulation and Test Results of Sealing System Reaction Force (Page 23)

| vehicle model | Average Test Value (N) | Reaction Force Converted to the Door Handle Position (N) | Sealing System Reaction Force (N) | Absolute Percentage Error APE | | | Mean Absolute Error MAE(N) | | Mean Absolute Percentage Error MAPE | | Root Mean Square ErrorRMSE（N） | |
| --- | --- | --- | --- | --- | --- | --- | --- | --- | --- | --- | --- | --- |
| Sedan A | 247±13 | 234 | 222 | | 5% | 25 | | 7% | | 18 | |  |
| SUV B | 252±13 | 210 | 226 | | 8% | 26 | | 8% | | 21 | |  |
| SUV C | 231±6 | 201 | 182 | | 9% | 49 | | 9% | | 20 | |  |

**C1-8:** Language, typos, and technical terms.

**A1-8:** Thank you very much for your suggestions.

The exaggerated adjectives and word spelling issues in this paper have been rechecked and revised.

The following sentence in Chapter 2.3 has been modified as follows: Hinges were modeled in such a way that the rotational degrees of freedom were released using BRE2RBE2 elements.

The content in Line 12 of the citation — "This computational approach quantifies the sequential load-deformation relationships between the seal strip and door structure, revealing transient force redistribution patterns not observable in static analysis" — has been deleted.

The content in Line 19 of the introduction — "The developed framework represents a significant advancement over prior art by systematically integrating multibody dynamics, nonlinear contact mechanics, and parametric sensitivity analysis within a unified computational platform" — has been deleted.

**C1-9:** PLOS ONE compliance.

**A1-9:** Thank you very much for your suggestions.

To ensure data usability, the door sealing system reaction force analysis model has been shared in the repository, including CLD simulation/test reports and boundary scripts.

**Minor revisions:**

**C2-1:** Title & author block

**A2-1:** Thank you very much for your suggestions.

To ensure data usability, the door sealing system reaction force analysis model has been shared in the repository, including CLD simulation/test reports and boundary scripts.

The title has been revised to "Prediction Method for the Reaction Force of Vehicle Door Sealing Systems" based on the research content. Page 1

**C2-2:** Abstract

**A2-2:** Thank you very much for your suggestions.

Replace "The error is within 10%" with "For the doors of three vehicle models (with n=3 for each model), the Mean Absolute Percentage Error (MAPE) between the simulated and predicted static closing force and the measured value is within 7%". See the details in the abstract section below:

The accuracy of the method for calculating the reaction force of the sealing system based on formula for moment equilibrium in statics is related to the degree of simplification of the mathematical model. Here, we demonstrate that the adoption of the finite element modeling method can fully consider the mutual coupling effect between the vehicle door and the flexible body of the sealing strip, eliminate errors caused by model simplification and single boundary variables, and improve the accuracy of predicting the reaction force of the sealing system. Contribution analysis can effectively identify the influencing factors of different regions of the sealing strip. In this study, a multi-boundary coupling simulation method for the door-sealing system was established based on the finite element theory, taking into account the interaction between the sealing strip and the complex structure of the door. This simulation method overcomes the limitations of single variables and simplified models in Excel calculations. The results show that for the doors of three vehicle models (with n=3 for each model), the Mean Absolute Percentage Error (MAPE) between the static closing force predicted by simulation and the actual measured value is 7%, which verifies the reliability of this method. Compared with the simplified calculation method of Excel, this simulation method can predict the reaction force of the door sealing system more accurately and intuitively. Our results demonstrate a more comprehensive strategy for predicting and optimizing the reaction force of the sealing system, which indicates that when the CLD of the sealing strip on the door lock side is increased by 50%, the total reaction force of the sealing system is increased by xx%. Page 3

**C2-3:** Define CLD at first mention.

**A2-3:** Thank you very much for your suggestions.

The definition of the sealing strip's CLD has been added; see Chapter 1.4 for details. Specifically, "The CLD of the sealing strip refers to Compression Load Deflection, which describes the relationship between the reaction force (unit: F) generated by a sealing strip sample (a 100mm-long cut sample) when compressed and the compression amount (unit: mm)." Page 13

Avoid "significant advancement" unless contrasted against a baseline with statistics.Avoid using "significant advancement" unless compared with a baseline supported by statistical data.

Response: Thank you very much for your suggestion. The content in Line 19 of the introduction — "The developed framework represents a significant advancement over prior art by systematically integrating multibody dynamics, nonlinear contact mechanics, and parametric sensitivity analysis within a unified computational platform" — has been deleted.

**C2-4:** The last line of the abstract

**A2-4:** Thank you very much for your suggestions.

The abstract has been revised as follows:

The accuracy of the method for calculating the reaction force of the sealing system based on formula for moment equilibrium in statics is related to the degree of simplification of the mathematical model. Here, we demonstrate that the adoption of the finite element modeling method can fully consider the mutual coupling effect between the vehicle door and the flexible body of the sealing strip, eliminate errors caused by model simplification and single boundary variables, and improve the accuracy of predicting the reaction force of the sealing system. Contribution analysis can effectively identify the influencing factors of different regions of the sealing strip. In this study, a multi-boundary coupling simulation method for the door-sealing system was established based on the finite element theory, taking into account the interaction between the sealing strip and the complex structure of the door. This simulation method overcomes the limitations of single variables and simplified models in Excel calculations. The results show that for the doors of three vehicle models (with n=3 for each model), the Mean Absolute Percentage Error (MAPE) between the static closing force predicted by simulation and the actual measured value is 7%, which verifies the reliability of this method. Compared with the simplified calculation method of Excel, this simulation method can predict the reaction force of the door sealing system more accurately and intuitively. Our results demonstrate a more comprehensive strategy for predicting and optimizing the reaction force of the sealing system, which indicates that when the CLD of the sealing strip on the door lock side is increased by 50%, the total reaction force of the sealing system is increased by xx%. Page 2

Based on the research content presented in the abstract and the full text, the keyword "Multi-boundary Coupling" has been replaced with "Nonlinear Finite Element". Additionally, the keyword "Accuracy" has been replaced with "NVH" (a standard abbreviation for Noise, Vibration, and Harshness in automotive engineering, retained in its original form without further translation).

**C2-5:** Introduction

**A2-5:** Thank you very much for your suggestions.

Content irrelevant to the sealing reaction force has been deleted from the introduction.

The revised content is as follows:

With the rapid electrification of the automotive industry, consumers have an increasing demand for refined driving experiences, which highlights the importance of vehicle NVH (Noise, Vibration, and Harshness) performance. Previous studies [1-3] have shown that the sound quality of vehicle door opening and closing has a significant impact on user perception and product satisfaction, and is a key influencing factor of vehicle NVH performance. The reaction force of the sealing system is an important evaluation index for the sound quality of door opening and closing, and directly affects the sound quality of door operation. Insufficient reaction force of the sealing system will weaken the sealing contact between the door and the vehicle body frame, leading to door vibration and dynamic sealing issues [4]; conversely, excessive reaction force of the sealing system will not only increase the door closing effort and amplify the unlocking sound (thereby reducing acoustic comfort), but also impose excessive mechanical stress on the locking system [5-9]. Reasonable prediction and control of the sealing system's reaction force have become an urgent task for automotive engineers, who aim to maintain a competitive advantage by enhancing user perception and satisfaction. This study intends to systematically analyze the reaction force of the NEV (New Energy Vehicle) door sealing system through simulation methods and propose innovative optimization strategies to improve the sound quality of door opening and closing. Wagner et al. [10] took the lead in conducting a nonlinear analysis of the seal compression load. Wang Jin et al. [11] established a mathematical model based on Excel to calculate the door closing force. Essentially, this calculation method simplifies the door and sealing system into a single, decoupled model, ignoring the interaction between the door and the seal, which leads to errors in the calculation results.

To overcome the aforementioned influencing factors, this paper proposes a multi-boundary coupling simulation method for the door sealing system. This method uses finite element modeling technology to establish a door-seal finite element model, and maps the measured/virtual CLD (Compression Load Deflection) curves to the assembled vehicle FE (Finite Element) model. The model considers the deformation of the door and the seal under flexible coupling, enabling accurate simulation of the sealing reaction force under closing conditions. In addition, this study implements a seal CLD value optimization strategy through contribution analysis, laying a foundation for the optimization of reaction force and the structural design of the seal. Meanwhile, tests were conducted on three vehicle models to verify the effectiveness and universality of the method. Page 3 and 6

**C2-6:** Literature gap

**A2-6:** Thank you very much for your suggestions.

In the introduction section, the reference "Prediction of Automotive Side Swing Door Closing" has been included. This reference indicates that previous engineers did not consider the flexible effect of vehicle doors. Addressing this issue, this study takes into account the flexible effect of vehicle doors and the coupling effect between the sealing strip and the door. The revised introduction is as follows:

With the rapid electrification of the automotive industry, consumers have an increasing demand for refined driving experiences, which highlights the importance of vehicle NVH (Noise, Vibration, and Harshness) performance. Previous studies [1-3] have shown that the sound quality of vehicle door opening and closing exerts a significant impact on user perception and product satisfaction, and serves as a key factor influencing vehicle NVH performance. The reaction force of the sealing system is a crucial evaluation index for the sound quality of door operation, directly affecting the sound quality of door opening and closing. Insufficient reaction force of the sealing system will weaken the sealing contact between the door and the vehicle body frame, leading to door vibration and dynamic sealing issues [4]; conversely, excessive reaction force of the sealing system will not only increase the door closing effort and amplify the unlocking sound (thereby reducing acoustic comfort), but also impose excessive mechanical stress on the locking system [5-9]. Reasonable prediction and control of the sealing system's reaction force have become an urgent task for automotive engineers, who aim to maintain a competitive advantage by enhancing user perception and satisfaction. This study intends to systematically analyze the reaction force of the NEV (New Energy Vehicle) door sealing system through simulation methods and propose innovative optimization strategies to improve the sound quality of door opening and closing. Wagner et al. [10] took the lead in conducting a nonlinear analysis of the seal compression load. Wang Jin et al. [11] established a mathematical model based on Excel to calculate the door closing force. Essentially, this calculation method simplifies the door and sealing system into a single, decoupled model, ignoring the interaction between the door and the seal, which results in errors in the calculation results.

To overcome the aforementioned influencing factors, this paper proposes a multi-boundary coupling simulation method for the door sealing system. This method utilizes finite element modeling technology to establish a door-seal finite element model and maps the measured/virtual CLD (Compression Load Deflection) curves to the assembled vehicle FE (Finite Element) model. The model considers the deformation of the door and the sealing strip under flexible coupling, enabling accurate simulation of the sealing reaction force under door-closing conditions. In addition, this study implements an optimization strategy for the CLD value of the sealing strip through contribution analysis, laying a foundation for the optimization of reaction force and the structural design of the sealing strip. Meanwhile, tests were conducted on three vehicle models to verify the effectiveness and universality of the proposed method. Page 3

**C2-7:** Correct typos:

**A2-7:** Thank you very much for your suggestions.

The content in Line 12 of the citation — "This computational approach quantifies the sequential load-deformation relationships between the seal strip and door structure, revealing transient force redistribution patterns not observable in static analysis" — has been deleted.

The following sentence in Chapter 2.3 has been modified as follows: Hinges were modeled in such a way that the rotational degrees of freedom were released using BRE2RBE2 elements.

**C2-8:** Claim calibration: Where you say “systematically overlooked,” add a citation or soften to “often neglected.”

**A2-8:** Thank you very much for your suggestions.

Additional citations have been added to the introduction to highlight the limitations of existing research.

With the rapid electrification of the automotive industry, consumers have an increasing demand for refined driving experiences, which underscores the importance of vehicle NVH (Noise, Vibration, and Harshness) performance. Previous studies [1-3] have indicated that the sound quality of vehicle door opening and closing significantly impacts user perception and product satisfaction, and serves as a key factor influencing vehicle NVH performance. The reaction force of the sealing system is a critical evaluation index for door operation sound quality, directly affecting the sound quality of door opening and closing. Insufficient reaction force of the sealing system weakens the sealing contact between the door and the vehicle body frame, leading to door vibration and dynamic sealing issues [4]; conversely, excessive reaction force of the sealing system not only increases door closing effort and amplifies the unlocking sound (thereby reducing acoustic comfort) but also imposes excessive mechanical stress on the locking system [5-9]. Reasonable prediction and control of the sealing system's reaction force have become an urgent task for automotive engineers, who aim to maintain a competitive edge by enhancing user perception and satisfaction. This study intends to systematically analyze the reaction force of the NEV (New Energy Vehicle) door sealing system through simulation methods and propose innovative optimization strategies to improve the sound quality of door opening and closing. Wagner et al. [10] pioneered the nonlinear analysis of seal compression loads.

ADD: Wang Jin et al. [11] established a mathematical model based on Excel to calculate door closing force. Essentially, this calculation method simplifies the door and sealing system into a single, decoupled model, ignoring the interaction between the door and the seal, which results in errors in the calculation results.

Page 3

**C2-9:** The sentence could be revised using something similar to the following.

“We therefore develop a multi-boundary, door–seal coupled model that maps measured/virtual CLD curves to an assembled vehicle FE model, accounts for door flexibility and contact, and quantifies regional contributions to the static closing force; we validate against bench tests on three vehicles.”

**A2-9:** Thank you very much for your suggestions.

The last paragraph of Chapter 1.3 has been revised as follows:

To address the aforementioned influencing factors, this paper proposes a multi-boundary coupling simulation method for the door sealing system. This method adopts finite element modeling technology to establish a door-sealing strip finite element model and maps the measured/virtual CLD (Compression Load Deflection) curves to the assembled vehicle FE (Finite Element) model. The model considers the deformation of the door and the sealing strip under flexible coupling, and can accurately simulate the sealing reaction force under closing conditions. In addition, this study implements an optimization strategy for the CLD value of the sealing strip through contribution analysis, laying a foundation for the optimization of reaction force and the structural design of the sealing strip. Meanwhile, tests are conducted on three vehicle models to verify the effectiveness and universality of the method. Page 6

**C2-10:** Theoretical Introduction (Pages 5-6)

Definition of static closing force: You defined it as the "minimum vertical force before the door lock engages". Ensure your tests adhere to this (your action of retracting the lock bolt is correct). Maintain consistency between the definition and the tests.

**A2-10:** Thank you very much for your suggestions.

Chapter 2.3 provides a detailed description of the test method, and the reaction force measured in the test is consistent with the definition of static reaction force in the theoretical section. The first paragraph of Chapter 4 has been revised as follows:

To verify the reliability and broad applicability of this analytical method, simulations and tests of the sealing system reaction force were conducted on three different vehicle models, including framed doors and frameless doors. The simulation analysis method is the same as that described in Chapter 3.1. The simulation analysis outputs the position of the door handle. The test results were first adjusted by subtracting the door lock force using Formula 2, then converted to the position of the door handle (the observation point in the simulation), and finally compared with the simulation results. Page 22

**C2-11:** Eq. (1)–(3) clarity Element types & mesh

**A2-11:** Thank you very much for your suggestions.

Equations 1–3 have been re-adjusted and re-derived, with a schematic diagram added. Symbols including Fj, Fmf, Fs, Fh, Fm, Fx, d0, F=Fm/100, L, Ls, and Ex(Is it E?) have been removed. The redefinition and description of formula symbols have also been deleted. For details, see Chapter 2.1. The content is as follows:

As shown in Figure 2, the vehicle door rotates around the hinge axis, and the sealing strip can be divided into several continuous segments. The moment arm from the i-th segment of the sealing strip to the hinge can be denoted as Li. Without considering door deformation and sealing strip compression deformation, the total moment of the sealing strip acting on the hinge axis is:

$E_{\text{md}}=\sum_{i=1}^{N} \text{F}_{i}\text{L}_{i}$ (1)

In the formula:

Emd is the total moment, with the unit of N·mm;

I is the total number of segments of the sealing strip;

Fi is the force acting on the i-th segment of the sealing strip, with the unit of N;

Li is the moment arm from the centroid of the i-th segment of the sealing strip to the hinge axis, with the unit of mm.

Then, according to the principle of moment equilibrium, the force at any point on the door relative to the hinge axis is:

$F=E_{\text{md}}/L_{s}=\frac{\left( \sum_{i=1}^{N} \text{F}_{i}\text{L}_{i} \right)}{L_{s}}$ (2)

Figure 2

Among them, Ls is the moment arm from any point on the door to the door hinge axis, with the unit of mm. The static closing force of the door is mainly contributed by three components: door seal, door latch mechanism, and buffer block. Page 6 and 7

（a）

（b）

**FIG. 4.** coupled FE model:（a）sealing strip（b）hinge page11

Figure 4 has been revised to [depict/show] the coupled FE model

Figures 1–3 have been merged into a single Figure 1. Figure 1(a) illustrates and labels four "functional zones", while Figure 1(c) shows the relative contact position between the sealing strip and the door, i.e., the position where the reaction force is applied by the sealing strip. The positions used to evaluate the contribution are consistent with the four "functional zones" of the sealing strip.

The door sheet metal structure is modeled using shell elements. For thin-walled sheet metal structures, the Kirchhoff shell theory is adopted, with the element property set to PSHELL. The solver used is NASTRAN SOL 106. The average mesh size of the door sheet metal is 8 mm, and the mesh quality complies with the internal corporate standards. Generally, a smaller mesh size leads to longer calculation time, while an excessively small mesh size affects calculation accuracy. This study uses an average mesh size of 8 mm, which balances the requirements for calculation efficiency and convergence.

**C2-12:** Seal representation

**A2-12:** Thank you very much for your suggestions.

The CBUSH spring element is selected for sealing strip simulation. The simulation is performed at intervals of 100 mm, and the measured/simulated force-displacement curves of the sealing strip are mapped to the CBUSH elements.

The CBUSH element is a spring-damper component capable of simulating stiffness in six directions. The orientation of the CBUSH element is determined based on the compression direction in the test, which corresponds to the Y-direction of the global coordinate system. Meanwhile, the CLD (Compression Load Deflection) force-displacement curve is assigned to the CBUSH element, as shown in the figure below:

**C2-13:** RBE2/RBE3 usage: “Bolts simulated by RBE2” acceptable, note any pretension (0 if none). / Hinges … rotational DOF released using RBE2” clarify which DOFs are constrained and how the hinge axis is defined. / Check and correct “BRE2” typos.

**A2-13:** Thank you very much for your suggestions.

Bolts represent a rigid connection relationship. Since the contact between bolts and their surrounding components as well as the preload force are ignored, the RBE2 rigid elements are used for simulation.

Hinges connect the door to the vehicle body and ensure the door rotates during opening and closing. As a revolute joint that rotates around a rotational axis, all degrees of freedom of the hinge are constrained, while the RBE2 element is used to release the hinge's rotational freedom around the axis for rotational movement.

The following sentence in Chapter 2.3 has been revised as:Hinges were modeled in such a way that the rotational degrees of freedom were released using BRE2RBE2 elements.

**C2-14:** Materials (Table 3): Units look like T/mm3 for density. Consider switching to kg/m3 for readability. Explain the adhesive modulus (1.63 MPa seems very low for a structural adhesive; if it’s a foam/tape, precise it).

**A2-14:** Thank you very much for your suggestions.

To enhance the readability of results, non-SI units are commonly used in finite element analysis. Specifically, the unit of length is mm (millimeter), the unit of mass is T (ton), and the units of stress/elastic modulus are MPa (megapascal). Regardless of the units employed, it is crucial to maintain unit consistency throughout the entire analysis process.

The density unit has been revised in accordance with the International System of Units (SI). The results are presented in the table below:

| Material | Elastic Modulus (MPa) | Poisson's Ratio | Density (KG/m³) |
| --- | --- | --- | --- |
| Steel | 206000 | 0.3 | 7830 |
| Spot Weld | 206000 | 0.3 | 7830 |
| Expansion glue | 1.63 | 0.41 | 1400 |

The expression of "adhesive" here is inaccurate; what is referred to is actually expanding glue. Therefore, the "adhesive material" section in the table has been revised to "expanding glue". Page 12

**C2-15:** Boundary conditions: Describe body-in-white constraints (are sills/hinge pillars fully fixed?), gravity on/off, and whether glass/regulators are included.

**A2-15:** Thank you very much for your suggestions.

A description of boundary conditions has been added to Chapter 2.

After establishing the multi-boundary coupling finite element model of the door-sealing strip, boundary conditions are applied to the model. The degrees of freedom of the mounting points at the joints between the upper and lower hinges and the vehicle body are constrained, while the hinges are allowed to rotate along the hinge axis. A Y-direction constraint is applied at the door handle position, and the NASTRAN SOL 106 nonlinear solver is used to simulate the stress state of the sealing strip during compression when the door is closed, and analyze the reaction force at the door handle, i.e., the sealing system reaction force. Page 17

The mounting points at the joints between the upper and lower hinges and the vehicle body are fully fixed, allowing the hinges to rotate along the hinge axis, while the Z-direction force due to gravity is not considered. The sealing system reaction force generated by the entire door is contributed solely by the sealing strip. For framed doors, the glass does not come into direct contact with the sealing strip, so the glass is not included in the finite element model; for frameless doors, the glass is in direct contact with the sealing strip, so the glass must be considered. The window regulator provides stiffness to the door; the model is simplified to include the regulator, but the internal motion relationships of the regulator are not considered.

**C2-16:** Workflow (Fig. 6): Add solver branch details (contact, iteration, checks).

Workflow (Figure 6): Add detailed information for the solver branch (contact, iteration, inspection).

**A2-16:** Thank you very much for your suggestions.

**C2-17:** Segment calibration for CLD (pp. 11–13)

**A2-17:** Thank you very much for your suggestions.

There have been significant revisions to the content of this section. In accordance with the feedback comments, a definition of the sealing strip CLD has been added to the first paragraph as follows:

The CLD (Compression Load Deflection) of a sealing strip, also referred to as "compression load-deflection", describes the relationship between the reaction force generated by the sealing strip when compressed and the amount of compression.

Next, the target values of key CLD parameters for the sealing strip in a specific project are introduced. The key parameters were obtained through cross-sectional simulation and testing, which verifies the accuracy of the simulation.

Method 1: Simulation

The simulation conditions are as follows:

Boundary Conditions: Extract the cross-section of the sealing strip, and set the length to 100 mm along the longitudinal direction in accordance with test standards. Use a solid mesh, and select the Mooney–Rivlin constitutive model for the material model. Adjust the mesh model to the uncompressed state of the sealing strip before assembly, and record the displacement S when the sealing strip returns to this uncompressed state.

Loading: Pre-assembly of the sealing strip: Apply fixed constraints to the cross-sections of the sheet metal and interior trim components; move the metal framework of the sealing strip and apply a forced displacement S along the assembly direction to complete the assembly.

Compression of the sealing strip: Apply fixed constraints to the sealing strip framework, side wall sheet metal and interior trim panel; move the door sheet metal and apply a specific forced angle along the compression direction of the sealing strip to achieve the required target compression amount.

Contact Type and Solver Settings: Adopt surface-to-surface contact. The surface normal behavior follows the penalty method, with a friction coefficient of 0.15 and no damping. The minimum load increment is 10−8, and an implicit solver is used. The software calculates the force residual, displacement correction, etc., for each load step, and compares these values with the convergence criteria. If the calculation results fall within the range of the criteria, the equilibrium iteration is considered convergent.

Fitting of Mooney–Rivlin/Ogden Models

Obtain test data of the rubber material, such as uniaxial test data, biaxial test data, and planar test data;

Establish a hyperelastic material in Abaqus, and input the test data using the "Test Data" option in the material editor;

Select the rubber material in the material editor, use the "Evaluate" tool, choose the corresponding test for fitting, and obtain the required constitutive parameters.

How to Export Region-Specific Tables

Export two curves through the output function: one is the curve of force vs. increment step, and the other is the curve of displacement vs. increment step. Process and convert these two curves into a single force-displacement (compression amount) curve, which is the required CLD curve. Set the displacement range as needed (e.g., -2 mm to +2 mm). The sealing strip compression amount D0 refers to the compression amount of the sealing strip when the door is closed, with the unit of mm.

Method 2: Testing Process

As follows (Page 16):At room temperature (23 ± 2) °C, take one 100 ± 1 mm sealing strip sample from each test position along the longitudinal direction of the sealing strip (see Figure 8). Install the sample on the test bench and place it at the center of the lower platform to ensure no pre-compression; adjust the upper pressure plate until it lightly touches the surface of the sample (with a contact force ≤ 0.1 N), and reset the displacement sensor; compress the sample at a speed of 5 mm/min until the target deformation is reached, and maintain the target deformation for 10 seconds after reaching it; finally, test one sample three times and take the average value. Organize the test results into a report.

**C2-18:** Analysis Results

**A2-18:** Thank you very much for your suggestions.

The constraint position of the handle is set at the centroid of the outer door handle assembly, with the coordinates as follows. The moment arm from this position to the hinge axis is 839 mm. The door and sealing strip are in their design states, and the door is assumed to be in the closed position by default. The sealing strip is in a compressed state under positive pressure, i.e., at the *D*0 position.

**C2-19:** “182 N at the handle”: Provide context; vehicle, configuration, and comparison to test value. Include a reaction-force vs. displacement curve rather than a single value. Report numerical tolerances at convergence.

**A2-19:** Thank you very much for your suggestions.

The analysis takes a specific SUV project as an example, where the front door is simulated and the results are correlated with test data. This analysis is a nonlinear static analysis. The output force is a single value at the observation point. The correlation results are presented in Table 7.

Table 7 Simulation and Test Results of Sealing System Reaction Force

| vehicle model | Average Test Value (N) | Reaction Force Converted to the Door Handle Position (N) | Sealing System Reaction Force (N) | Absolute Percentage Error APE | | | Mean Absolute Error MAE(N) | | Mean Absolute Percentage Error MAPE | | Root Mean Square ErrorRMSE（N） | |
| --- | --- | --- | --- | --- | --- | --- | --- | --- | --- | --- | --- | --- |
| Sedan A | 247±13 | 234 | 222 | | 5% | 25 | | 7% | | 18 | |  |
| SUV B | 252±13 | 210 | 226 | | 8% | 26 | | 8% | | 21 | |  |
| SUV C | 231±6 | 201 | 182 | | 9% | 49 | | 9% | | 20 | |  |

**C2-20:** Contours (Figs. 10-11): Add color bars with units (N for contact pressure/reaction, if it’s stress, specify MPa; for displacement, mm). Add views that show the four regions.

Contribution analysis (pp. 16–18). Clarify vehicle names or keep anonymized consistently (“Sedan A,” “SUV B”).

**A2-20:** Thank you very much for your suggestions.

Color bars and units have been added to Figures 10 and 11.

**FIG. 10.** Reaction force contour at the observation point

**FIG. 11.** Y-direction displacement contour of the door frame**(Page18 and 19)**

Since the spring elements of the sealing strip are modeled using 1D elements, the post-processing results for the four regions are presented as follows:

The three test vehicles remain anonymous, with their names revised from "a certain sedan", "a certain compact SUV", and "a certain full-size SUV" to "Sedan A", "SUV B", and "SUV C" respectively.

**C2-21:** Conclusion

**A2-21:** Thank you very much for your suggestions.

The revised conclusions are as follows.

Herein, we demonstrate that the sealing system reaction force simulation method can address the accuracy error issue caused by calculations using the simplified Excel mathematical model. Notably, this method accounts for the mutual coupling effect between the door and the flexible body of the sealing strip, as well as the influence of the sealing strip's deformation after compression on the reaction force. Taking the front door of a specific SUV as an example, an analytical method for the door sealing system reaction force was established. Meanwhile, we conducted a comparative study on the simulation and test results of the sealing system reaction force across three different vehicle models. The results show that the Mean Absolute Percentage Error (MAPE) of the three vehicles is 7%, with a maximum error of 9%, verifying the effectiveness and reliability of the proposed method.

Additionally, the analysis of the contribution of the sealing system reaction force yielded results regarding the impact of different regions of the sealing strip on the reaction force, based on which a segmented optimization strategy for the sealing strip was proposed. Furthermore, through the simulation and test data of the three different vehicle models, we found that this simulation method is applicable to both framed-door and frameless-door vehicle models. By predicting the sealing system reaction force, controlling it, and guiding the structural design of the sealing strip to meet the development requirements of different projects, this method exhibits portability and promotability.

However, this simulation method is only suitable for predicting the sealing system reaction force when the door is in the closed state; it cannot account for the dynamic process of door closing or the door-closing energy. The working conditions of the dynamic door-closing process are more complex, and the simulation methodology remains immature, which can be regarded as a direction for future research. (Page 2)

**C2-22:** Add one design takeaway (“raising latch-side CLD by 10% increases total closing force by xx% due to lever-arm dominance, suggesting targeted softening at latch to reduce effort without compromising seal at sill/header”).

**A2-22:** Thank you very much for your suggestions.

Added herein are key design points: the CLD curve of the door lock-side sealing strip has been improved by 50%, and the total sealing system reaction force has been increased by 19%.

**C2-23:** References (pp. 20–22)

**A2-23:** Thank you very much for your suggestions.

Irrelevant references have been deleted, and the remaining references have been formatted in a standardized way, following the order of author, year, article title, journal, volume (issue): pages, and DOI. Each DOI has also been verified. Thank you.

[1] Dongju Kim，Sangmin Jeon. et al. 2023.Strategies for Reducing Booming Noise Generated by theTailgate of an Electric Sport Utility Vehicle [J] . Applied Sciences,13(24).<https://doi.org/10.3390/app132413134>
[2] Ding Fei; Xie Wutong; Xie Xiaoping.2023.Research on Optimization of car door closing sound quality based on the integration of structural simulation and test.[J] . Volume 237 , Issue 6 .DOI:10.1177/09544070221088370
[3] Hamilton, D.1999."Sound Quality of Impulsive Noise: An Applied Study of Automotive Door Closing Sounds". SAE Technical Paper 1999-01-1684.
[4] Kun, E.Y. and Mhta, P.R. 2003，"The Effect of Seal Stiffness on Door Chucking and Squeak and Rattle Performance," SAE Technical Paper 2003-01-1521, <https://doi.org/10.4271/2004-01-1562>.
[5] Hyeonho Jo.Weonchan Sung et al. 2015.A Study on Developing Sound Quality Index of Car Door Latch and Improving Sound Quality by Changing Door Latch Assembly Design.[J] Transactions of the Korean Society for Noise and Vibration Engineering. Volume 25 , Issue 9 . PP 614-619
[6] Musser, C.T, 2005, "Application of Transient SEA for Vehicle Door Closure Sound Quality," SAE Technical Paper 2005-01-2433, <https://doi.org/10.4271/2005-01-2433>.
[7] Pang J, 2015, Control of Noise and Vibration of Vehicle Body, Beijing: China Machine Press.
[8] Kim, S.-Y., Ryu, S.-C., Jun, Y.-D., et al.2022, "Methodology for Sound Quality Analysis of Motors for Automotive Interior Parts through Subjective Evaluation," Sensors, vol. 22, no. 18, p. 6898, Sep, <https://doi.org/10.3390/s22186898>.
[9] Yalcin, E., Bilal, H., Yagci, A., et al., "A Numerical Approach for Sound Quality of Vehicle Doors," International Journal of Acoustics and Vibration, vol. 25, no. 1, pp. 9-16, Mar. 2020, <https://doi.org/10.20855/ijav.2020.25.11475>.
[10] Wagner, D.A., Morman, K.N., Gur, Y., et al. 1997, "Nonlinear Analysis of Automotive Door Weatherstrip Seals," Finite Elements in Analysis and Design, vol. 28, no. 1, pp. 33-50.
[11] Jing Li , Zissimos P. Mourelatos 2009, "Prediction of Automotive Side Swing Door Closing Effort"SAE Technical Paper 2009-01-0084.
[12] Huang, J.L.,Automotive Body Design, Beijing: China Machine Press, 2007.
[13] Liu, Q.L. and Li, H.,Theoretical Mechanics, Beijing: Science Press, 2005.
[14] Gu, X., "A Brief Discussion on the Design of Automotive Rubber Sealing Strips,"Internal Combustion Engine & Parts, vol. 2018, no. 17, 2018, https://doi.org/10.19475/j.cnki.issn1674-957x. 2018.17.030. **(Page 25 and 28)**

**Answers to Reviewer(1):**

**C3-1:** Title

**A3-1:** Thank you very much for your suggestions.

This paper focuses on the simulation and prediction method for the reaction force of sealing systems. Therefore, to highlight the core focus, the title of this paper has been revised based on the research content to:

Prediction Method for the Reaction Force of Vehicle Door Sealing Systems

(Page1)

**C3-2:** The abstract lacks consideration of logical coherence. Please add research background, such as the deficiency in sealing reactive force prediction and breakthrough points in measurement method.

**A3-2:** Thank you very much for your suggestions.

The abstract has been supplemented with content on current reaction force calculation methods and their shortcomings; based on this, the innovative research carried out in this paper is also incorporated. The revised abstract is as follows:

The accuracy of the method for calculating the sealing system reaction force based on formula for moment equilibrium in statics is related to the degree of simplification of the mathematical model. Herein, we demonstrate that the adoption of the finite element modeling method can fully account for the mutual coupling effect between the door and the flexible seal, eliminate errors caused by model simplification and single boundary variables, and improve the accuracy of sealing system reaction force prediction. Contribution analysis can effectively identify the influencing factors of different seal regions.In this study, a multi-boundary coupling simulation method for the door-sealing system was established using finite element theory, which considers the interaction between the seal and the complex door structure. This simulation method overcomes the limitations of Excel calculations, such as single variables and simplified models. The results show that, among the doors of three vehicle models (with n=3 for each model), the mean absolute percentage error (MAPE) between the static closing force predicted by simulation and the actual measured value is 7%—verifying the reliability of this method. Compared with the simplified calculation method using Excel, this simulation method can predict the door sealing system reaction force more accurately and intuitively.Our results present a more comprehensive prediction and optimization strategy for the sealing system reaction force. This strategy shows that a 75% increase in the CLD (Compression Load Deflection) of the lock-side seal leads to a 22% increase in the total sealing system reaction force.

(Page 2)

**C3-3:** The image is not clear enough (such as Fig2), please redraw this Fig and include necessary annotations to ensure clarity and completeness.

**A3-3:** Thank you very much for your suggestions.

Figure 2 has been reorganized and redrawn, and merged with other figures into Figure 1. Corresponding descriptions have also been added, which can be found in Section 1.2.

**Fig. 1. (a)**Schematic Diagram of the Door Sealing System**(b)** Cross section of a typical vehicle door seal installation**(c)** Exploded View of the Door-Seal System

The door skin and side outer panel establish direct contact with the seal, as presented in the assembly cross-section Figure 1(b) and exploded view in Figure 1(c). Figure 1(c)

It reflects the relative contact position between the sealing strip and the door, i.e., the position where the sealing strip exerts the reaction force. (Page 8 and 9)

**C3-4:** The lacks consideration of the relationship between Fig3 a b and c; please provide additional commentary on the meaning of the curves in Fig3 c to enhance clarity and understanding.

**A3-4:** Thank you very much for your suggestions.

Section 2.4 "Sealing Strip Calibration" has been fully updated. Figures 3a, 3b, and 3c all show simulation-derived CLD curves, with only the compression amount on the horizontal axis differing. Therefore, the three graphs have been integrated into two. These are shown below:

FIG. 7. CLD Curve of Door Frame Seal

FIG. 8. CLD Curve of Door Seal**(Page15and16)**

**C3-5:** In Section 2.2, the input sources for the sealing strip parameters are not specified. Please provide specific methods and references for obtaining these parameters and explain the rationale and accuracy of the parameter selection.

**A3-5:** Thank you very much for your suggestions.

The parameters of the sealing strip are obtained through simulation/testing.

**Method 1: Simulation**

**The working conditions are as follows:**

**Boundary conditions:**

**Cut the cross-section of the sealing strip, take a 100 mm length unit along the length direction in accordance with test standards, use a solid mesh, and select the Mooney–Rivlin constitutive model for the material model.**

**Adjust the mesh model to the uncompressed state of the sealing strip before assembly, and record the displacement S when the sealing strip returns to this uncompressed state.**

**Loading:Pre-assembly of the sealing strip: Fix and constrain the cross-sections of sheet metal and interior trim components, move the metal skeleton of the sealing strip, and apply a forced displacement S along the assembly direction to achieve the assembly purpose.**

**Compression of the sealing strip: Fix and constrain the sealing strip skeleton, side wall sheet metal, and interior trim panel, move the door sheet metal, and apply a certain forced angle along the sealing strip compression direction to reach the required target compression amount.**

**Contact type and solver settings:**

**Use surface-to-surface contact; the surface normal behavior is set to penalty, the friction coefficient is 0.15, and there is no damping. The minimum load increment is 1e-8, and an implicit solver is used.**

**The software calculates force residuals, displacement corrections, etc., for each load step, and compares them with the convergence criteria. If the calculation results fall within the criteria, the equilibrium iteration is considered converged.**

**Method 2: Test Process**

**The test on the sealing strip sample is conducted in accordance with QC/T 710-2004 "Test Method for Compression Load of Automotive Sealing Strips":**

**At room temperature (23±2℃), obtain a 100±1 mm segment of the sealing strip sample from the test position along the length direction of the sealing strip (see Figure 8).**

**Install the sample on the tooling bench and place it in the center of the lower platform to ensure no pre-compression.**

**Adjust the upper pressure plate to lightly touch the surface of the sample (with a contact force ≤ 0.1 N), and reset the displacement sensor.**

**Compress the sample at a speed of 5 mm/min until the target deformation is reached, and maintain the target deformation for 10 seconds after reaching it.**

**Finally, conduct the test on the sample 3 times and take the average value.**

**Organize the test results into a report, and correlate them with the simulation results to ensure the reliability of the simulation analysis results.**

**C3-6:** In Section 2.2, please provide a detailed explanation of whether the compression load deflection (CLD) test for the sealing strips was conducted. If the test was performed, please clearly specify the specific methods and steps of the CLD test, including the experimental equipment used, test standards followed, and critical parameters involved. Additionally, please describe the process of sample preparation, including the number, type, and dimensions of the samples, to ensure the scientific rigor and logical consistency of the experimental methods .

**A3-6:** Thank you very much for your suggestions.

The compression load test for sealing strips has been added to Section 2.2, as follows: To ensure the accuracy of input parameters, a compression load test was conducted on the door weatherstrip and door frame weatherstrip . The test equipment and setup are shown in Figure 7:

Figure 7: Test Bench for Sealing Strip Compression Load

At room temperature (23±2℃), obtain one segment of 100±1 mm sealing strip sample from each test position along the length direction of the sealing strip (see Figure 8). Install the sample on the tooling bench and place it at the center of the lower platform to ensure no pre-compression; adjust the upper pressure plate to lightly touch the surface of the sample (with a contact force ≤ 0.1 N) and reset the displacement sensor; compress the sample at a speed of 5 mm/min until the target deformation is reached, and maintain the target deformation for 10 seconds after reaching it; finally, conduct three tests on one sample and take the average value.

Figure 8: Sealing Strip Test Specimen (Page 16)

**C3-7:** In Section 2.2, please provide the specific description of simulation method and extraction method of boundary.

**A3-7:** Thank you very much for your suggestions.

The simulation method and boundary conditions adopted in this paper are as follows:

Simulation Method: The NASTRAN SOL 106 solver is used to perform nonlinear static analysis on the door.

Boundary Conditions:

Constrain the degrees of freedom (DOFs) of the mounting points where the upper and lower hinges connect to the vehicle body, and release the rotational degree of freedom of the hinges along the hinge axis.

Apply a Y-direction constraint at the door handle position, map the CLD curve to the door, simulate the stress state of the sealing strip when compressed under the door-closed condition, and analyze the reaction force at the door handle—i.e., the sealing system reaction force. (Page 17)

**C3-8:** Section 2.3 please add test preparation, test method, test process and test data extraction, etc.

**A3-8:** Thank you very much for your suggestions.

To verify the reliability and broad applicability of the proposed analysis method, Chapter 4 conducts simulation and testing of the sealing system reaction force on three different vehicle models, including those with framed doors and frameless doors. The simulation analysis method is consistent with that described in Section 2.1, and the testing method aligns with the definition of static closing force.

The test equipment and setup are shown in Figure 12. Tests were conducted on the prototype vehicle to ensure its state is consistent with the designed state. Adjust the door to the closed position, but keep the door lock in the unlocked state. Use a dynamometer (accuracy: ±1 N) to slowly apply a Y-direction pushing force at the outer panel position corresponding to the door lock (30 mm away from the edge of the outer panel) until the door is locked. Record the dynamometer reading at this point as the door closing force; take the average value of three test results as the final test result and document it. A comparison between the simulation results and test results of the sealing system reaction force is shown in Table 5:

FIG. 13 Static Closing Force Test

Table 7: Comparison between Simulation and Test Results of Sealing System Reaction Force

| vehicle model | Average Test Value (N) | Reaction Force Converted to the Door Handle Position (N) | Sealing System Reaction Force (N) | Absolute Percentage Error APE | | | Mean Absolute Error MAE(N) | | Mean Absolute Percentage Error MAPE | | Root Mean Square ErrorRMSE（N） | |
| --- | --- | --- | --- | --- | --- | --- | --- | --- | --- | --- | --- | --- |
| Sedan A | 247±13 | 234 | 222 | | 5% | 25 | | 7% | | 18 | |  |
| SUV B | 252±13 | 210 | 226 | | 8% | 26 | | 8% | | 21 | |  |
| SUV C | 231±6 | 201 | 182 | | 9% | 49 | | 9% | | 20 | |  |

**C3-8:** In Section 3.3, The article lacks consideration of the description of test results. Please provide test and experimental validation. Is the verification and comparison between test results and simulation data consistent and representative? Please perform test and experimental validation on 3-5 sets of data and provide detailed explanations of the test results.

**A3-8:** Thank you very much for your suggestions.

Details of the sealing system reaction force test can be found in Section 3.3, and some wording has been adjusted. The specific adjustments are as follows:

To verify the reliability and broad applicability of this analysis method, simulation and testing of the sealing system reaction force were conducted on three different vehicle models, including those with framed doors and frameless doors. The simulation analysis method is consistent with that described in Section 2.1.

The test equipment and setup are shown in Figure 12. Tests were performed on the prototype vehicle to ensure its state matches the designed state. Adjust the door to the closed position while keeping the door lock in the unlocked state. Use a dynamometer (accuracy: ±1 N) to slowly apply a Y-direction pushing force at the outer panel position corresponding to the door lock (30 mm away from the edge of the outer panel) until the door is locked. Record the dynamometer reading at this point as the door closing force, then take the average of three test results as the final test result and document it. A comparison between the simulation results and test results of the sealing system reaction force is presented in Table 7:

Table 7: Comparison between Simulation and Test Results of Sealing System Reaction Force

| vehicle model | Average Test Value (N) | Reaction Force Converted to the Door Handle Position (N) | Sealing System Reaction Force (N) | Absolute Percentage Error APE | | | Mean Absolute Error MAE(N) | | Mean Absolute Percentage Error MAPE | | Root Mean Square ErrorRMSE（N） | |
| --- | --- | --- | --- | --- | --- | --- | --- | --- | --- | --- | --- | --- |
| Sedan A | 247±13 | 234 | 222 | | 5% | 25 | | 7% | | 18 | |  |
| SUV B | 252±13 | 210 | 226 | | 8% | 26 | | 8% | | 21 | |  |
| SUV C | 231±6 | 201 | 182 | | 9% | 49 | | 9% | | 20 | |  |

Figure 3

Sealing system reaction force tests were conducted on three vehicle models. The average test values were converted to the reaction force at the handle position for correlation analysis in accordance with Equations (1) and (2). The mean absolute percentage error (MAPE) between the sealing system reaction force simulation and the experiment was 7%, with a maximum error of 9%. A residual plot was generated based on the test results, which showed that the residuals were both positive and negative, fluctuating around zero. This confirms the consistency of data trends and indicates that the analysis results are reliable. Therefore, the door sealing system reaction force prediction method established in this study provides reliable methodological support for the design and optimization of sealing strips in the early stage of project development. (Page 24)

**C3-9:** The description of popularization and application is not sufficient. Please provide the application cases of the method in practical engineering, and discuss the application scope and potential application fields of the method.

**A3-9:** Thank you very much for your suggestions.

A discussion on the application scope has been added to the conclusions, as follows: Based on the simulation and test data of three different vehicle models, we found that this simulation method is applicable to both vehicles with framed doors and those with frameless doors. By predicting the sealing system reaction force, controlling the reaction force of the sealing system, and guiding the structural design of the sealing strip to meet the development needs of different projects, this method demonstrates portability and promotability. (Page 25)

**C3-10:** The conclusion of the thesis is presented in a decentralized manner. Please provide that after the above modification is complete, the conclusion of the thesis be revised, the core achievements and contributions of the research work be summarized, and the value of the research achievements in practical application be analyzed. Based on the research results, the possible future research directions or improvement measures be put forward to make the conclusion of the thesis more clear and complete and to fully show the value and significance of the research.

**A3-10:** Thank you very much for your suggestions.

I have completed the above revisions based on the suggestions provided. Additionally, to highlight the research achievements and demonstrate the value of the research results in practical applications, the logical structure and language of the conclusions have been revised. The revised conclusions are as follows:

Herein, we demonstrate that the sealing system reaction force simulation method can address the accuracy error caused by calculations using the simplified Excel mathematical model. Notably, this method accounts for the mutual coupling effect between the door and the flexible body of the sealing strip, as well as the influence of the sealing strip’s deformation after compression on the reaction force. Taking the front door of a specific SUV as an example, an analysis method for the door sealing system reaction force was established. Meanwhile, we conducted a comparative study on the simulation and test results of the sealing system reaction force for three different vehicle models. The results show that the simulation accuracy reaches 90%, verifying the effectiveness and reliability of the proposed method. Additionally, through the analysis of the contribution of the sealing system reaction force, we obtained results regarding the influence of different regions of the sealing strip on the reaction force, and based on this, proposed a segmented optimization strategy for the sealing strip. Furthermore, based on the simulation and test data of three different vehicle models, we found that this simulation method is applicable to vehicles with framed doors and frameless doors. By predicting the sealing system reaction force, controlling the sealing system reaction force, and guiding the structural design of the sealing strip to meet the development needs of different projects, this method exhibits portability and promotability. However, this simulation method is only suitable for predicting the sealing system reaction force when the door is in the closed state; it cannot account for the dynamic process of door closing or the door-closing energy. The working conditions of the dynamic door-closing process are more complex, and the simulation methods for this process are still immature, which can be considered a direction for future research. (Page 25)

| Position | | D0-2  N/100mm | D0  N/100mm | D0+2  N/100mm |
| --- | --- | --- | --- | --- |
| Door Strip N/100mm | Hinge | 4.5 | 5.42 | 6.13 |
|  | Roof Rail | 5.2 | 6.6 | 8.81 |
|  | Door Lock | 4.82 | 5.58 | 6.02 |
|  | Sill | 5.59 | 7.31 | 9.46 |
| Frame Strip N/100mm | Hinge | 0.83 | 4.37 | 7.09 |
|  | Roof Rail | 0.81 | 4.36 | 6.91 |
|  | Door Lock | 0.69 | 4.38 | 7.21 |
|  | Sill | 0.76 | 4.46 | 7.12 |
